# Supplementary material for: Reversible Diels–Alder Addition to Fullerenes: A Study of Dimethylanthracene with H2@C60
Source: Nanomaterials (Basel). 2022 May 13;12(10):1667. doi: 10.3390/nano12101667 (PMC9144212; doi:10.3390/nano12101667)
Supplement: Supplementary file 1 [file nanomaterials-12-01667-s001.zip › nanomaterials-1681349-supplementary.pdf]

Supplementary Information for

Reversible Diels–Alder Addition to Fullerenes: A Study of Dimethylanthracene with $H_2@C_{60}$

Mahboob Subhani [1,†], Jinrong Zhou [1,†], Yuguang Sui [1], Huijing Zou [2], Michael Frunzi [3], James Cross Jr. [3], Martin Saunders [3], Cijun Shuai [4,*], Wenjie Liang [1,*] and Hai Xu [1,5,*]

1 College of Chemistry and Chemical Engineering, Central South University, South Lushan Road, Changsha 410083, China; mehboobsubhani814@gmail.com (M.S.); 18171099526@163.com (J.Z.); sui_yg@csu.edu.cn (Y.S.)
2 Department of Biology, College of Arts and Science, New York University, New York, NY 10012-3, USA; hz2750@nyu.edu
3 Department of Chemistry, Yale University, New Haven, CT 06520, USA; micheal.frunzi@gmail.com (M.F.); james.cross@yale.edu (J.J.C.); ms@gaus90.chem.yale.edu (M.S.)
4 College of Mechanical and Electrical Engineering, Central South University South Lushan Road, Changsha 410083, China
5 Shenzhen Research Institute of Central South University, High-Tech Industrial Park, Yuehai Street, Shenzhen 518057, China
* Correspondence: shuai@csu.edu.cn (S.C.); liang_wenjie@163.com (W.L.); xhisaac@csu.edu.cn (X.H.)
† These authors contributed equally to this work.

This file includes:

Figure S1–S7

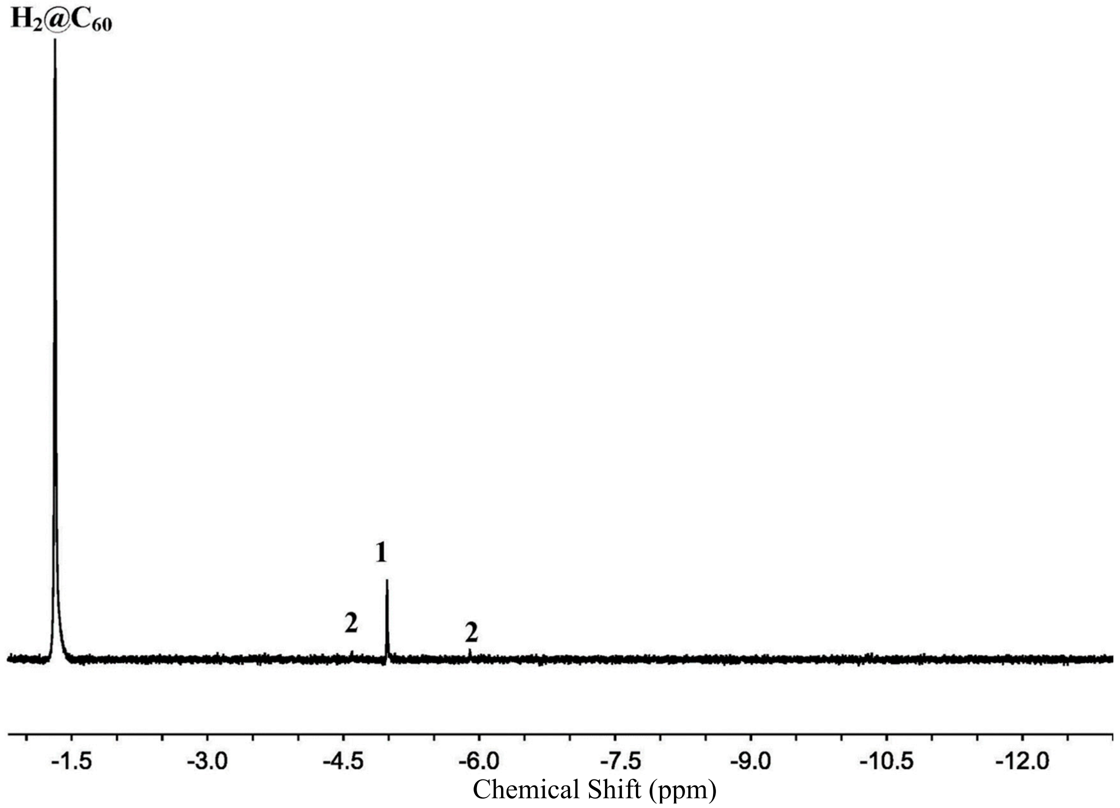

Figure S1.^{1}H NMR spectra of $H_2@C_{60}$ with 0.5 eq DMA

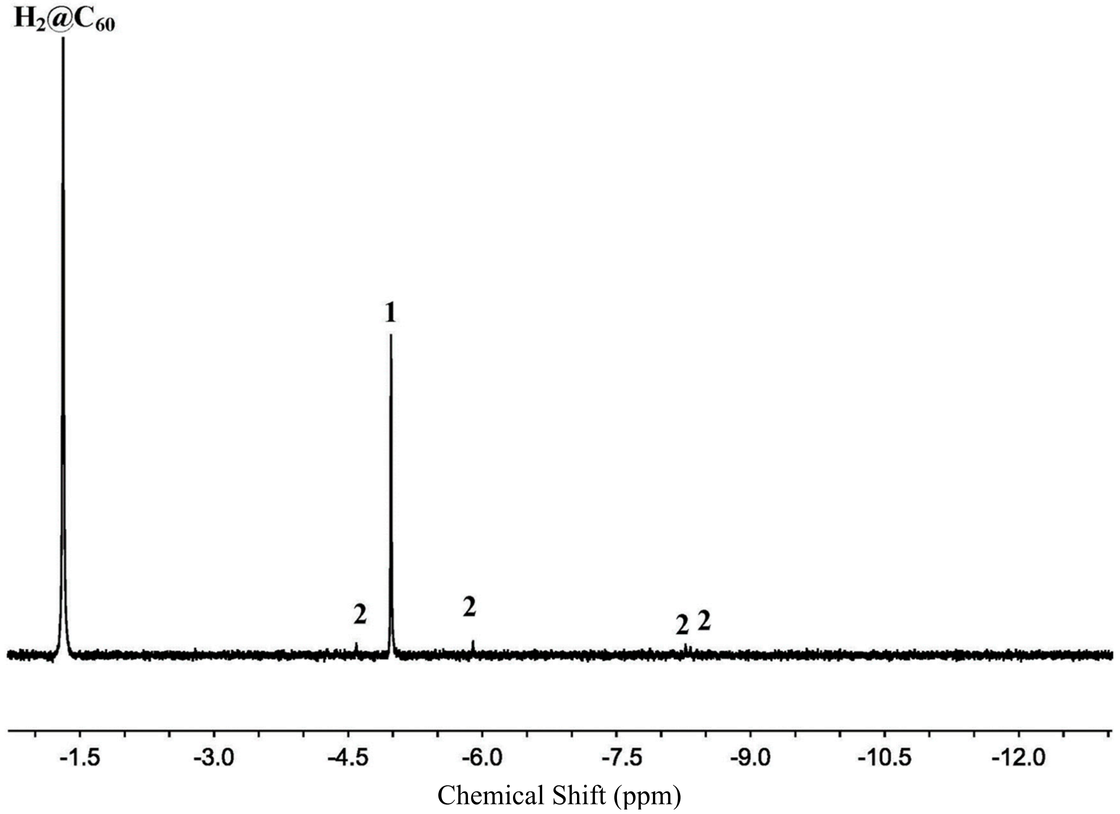

Figure S2.^{1}H NMR spectra of $H_2@C_{60}$ with 1 eq DMA

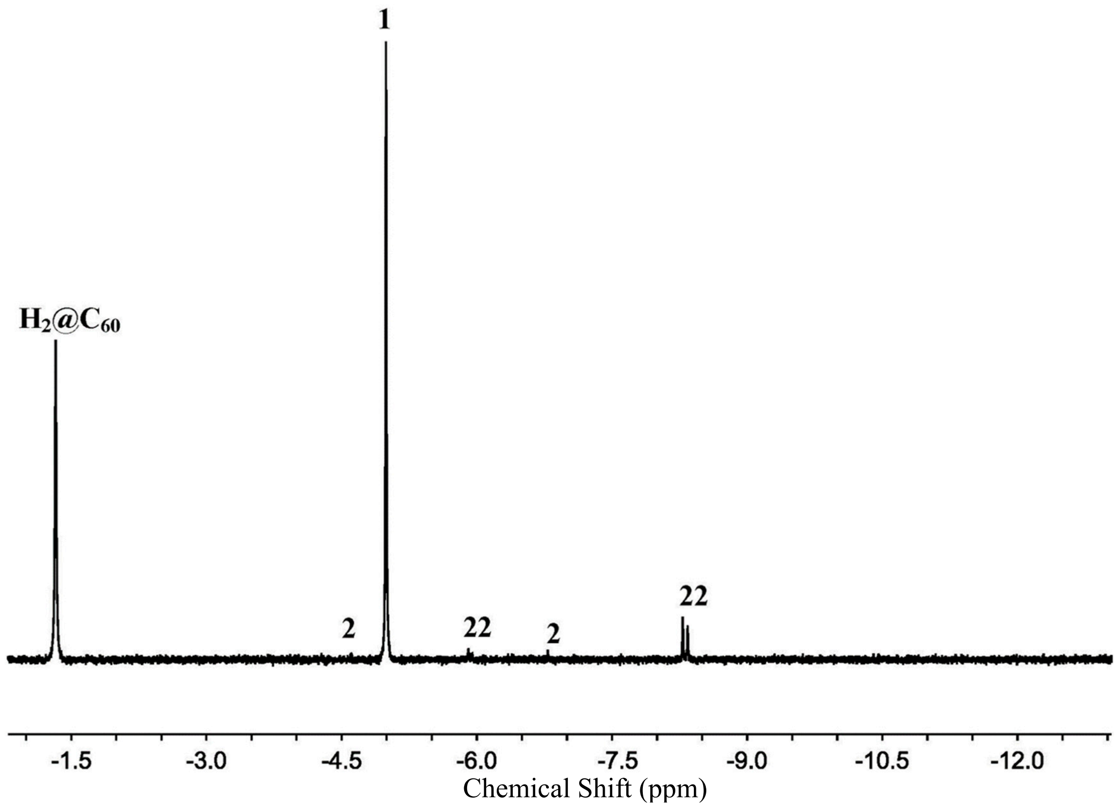

Figure S3.^{1}H NMR spectra of $H_2@C_{60}$ with 1.5 eq DMA

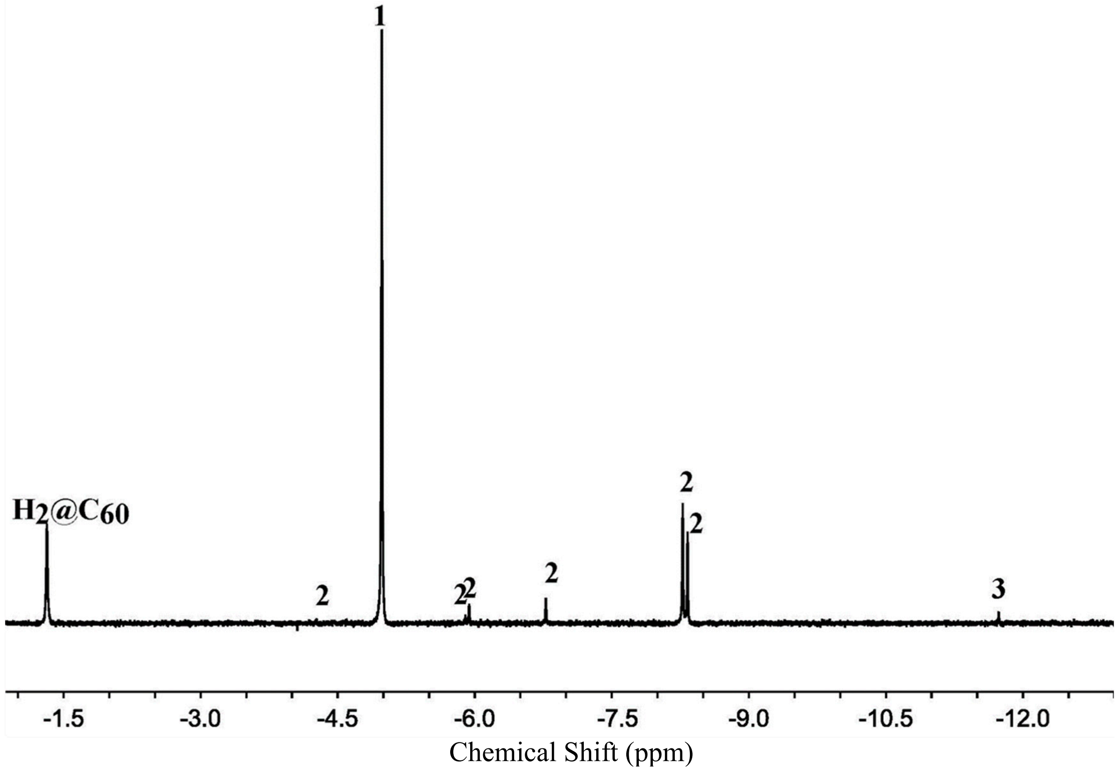

Figure S4.^{1}H NMR spectra of $H_2@C_{60}$ with 2 eq DMA

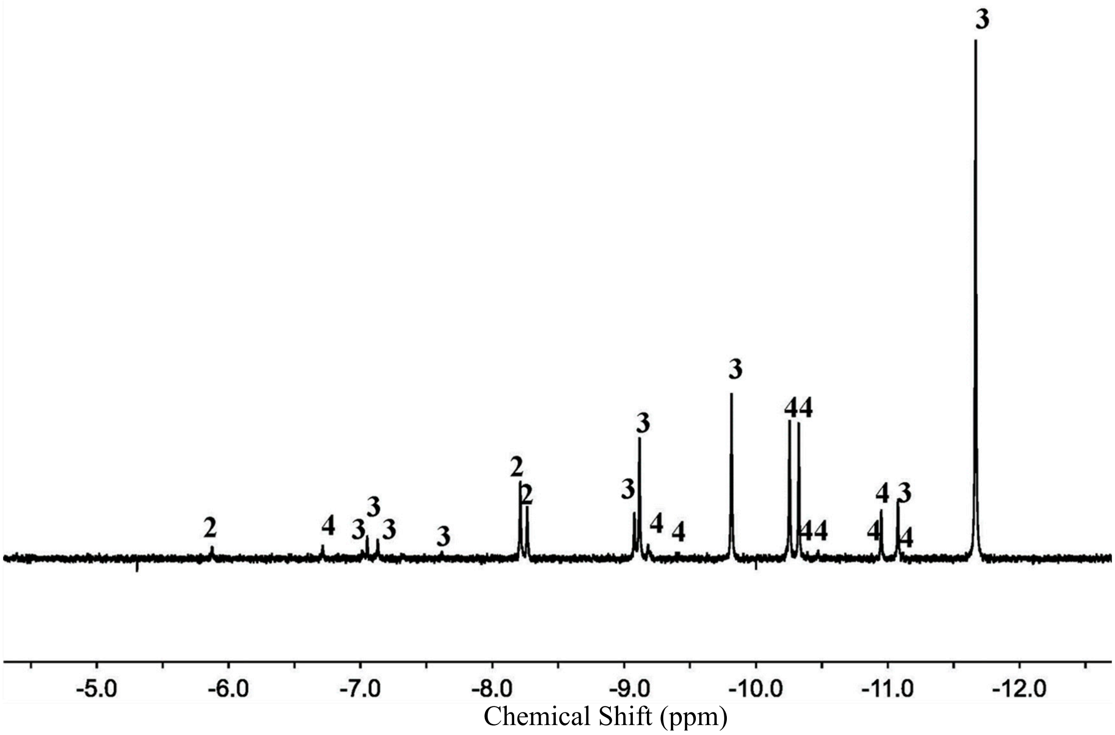

Figure S5.^{1}H NMR spectra of $H_2@C_{60}$ with 15 eq DMA

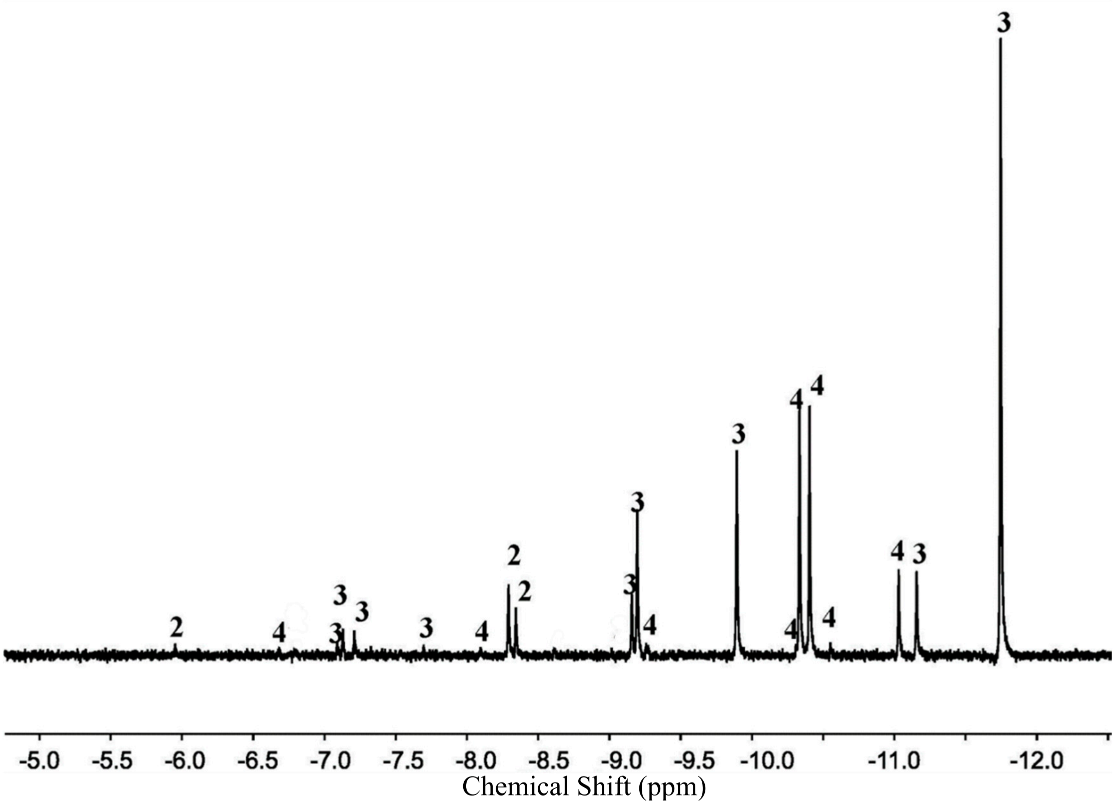

Figure S6.^{1}H NMR spectra of $H_2@C_{60}$ with 20 eq DMA

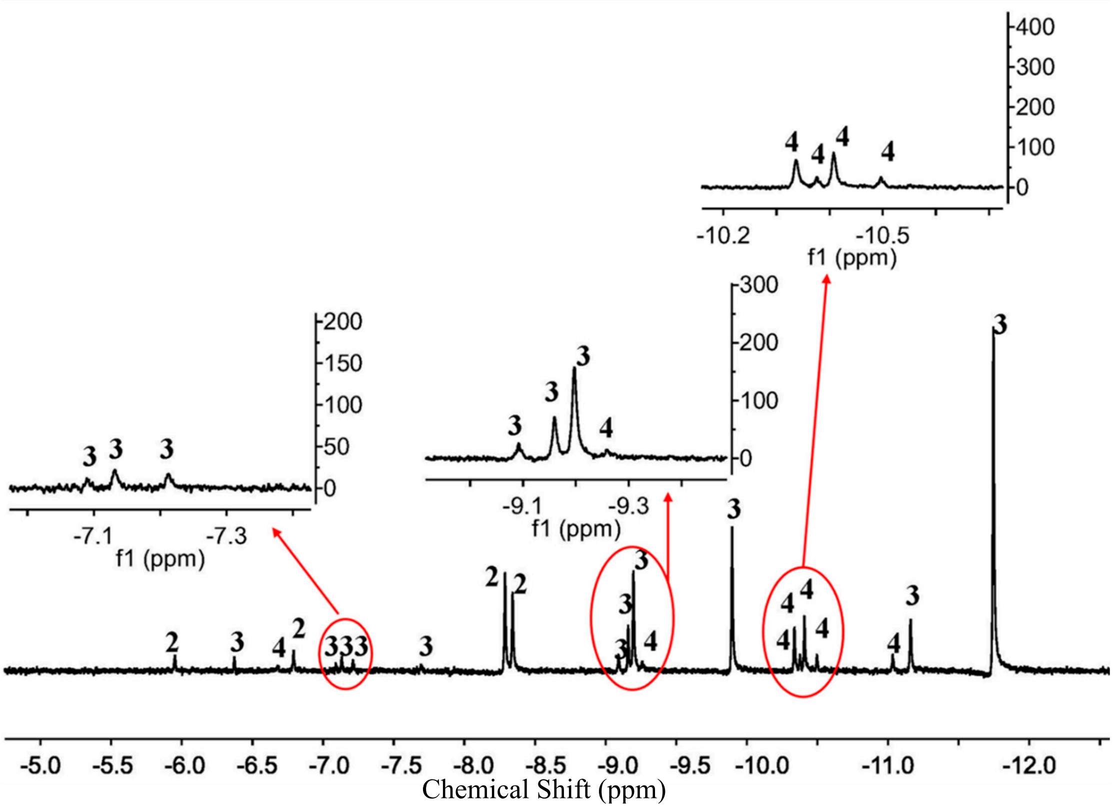

Figure S7.^{1}H NMR spectra of $H_2@C_{60}$ with 10 eq DMA
